# An anatomy-guided whole-heart framework for computational modelling of the complete human cardiac conduction system

**DOI:** 10.3389/fcvm.2026.1872859

**Published:** 2026-10-20

**Authors:** Mohammadreza Kariman, Christoph M. Augustin, Thomas Schrotter, Matthias A. F. Gsell, Edward J. Vigmond, Gernot Plank

**Affiliations:** 1 Gottfried Schatz Research Center, Division of Medical Physics and Biophysics, Medical University of Graz, Graz, Austria; 2 BioTechMed-Graz, Graz, Austria; 3 IHU Liryc, Electrophysiology and Heart Modeling Institute, Fondation Bordeaux Université, Liryc, Bordeaux, France

**Keywords:** cardiac conduction system, cardiac digital twin, computational modelling, His-Purkinje system, whole-heart electrophysiology

## Abstract

**Introduction:**

Computational models of cardiac electrophysiology are increasingly used to investigate arrhythmia mechanisms and evaluate therapeutic interventions, yet most existing frameworks omit or oversimplify the cardiac conduction system, limiting their physiological fidelity and clinical applicability.

**Methods:**

We present a subject-specific, anatomy-guided computational model of the complete human cardiac conduction system (CCS) integrated within a four-chamber heart and torso geometry derived from magnetic resonance imaging. The model comprises a three-dimensional sinoatrial node with discrete exit pathways, interatrial conduction pathways including Bachmann’s bundle and posterosuperior connections, an atrioventricular node with explicit fast and slow pathway anatomy, His bundle, left bundle branch with trifascicular configuration, right bundle branch, and fascicle-anchored Purkinje networks. The unknown ramification topology of the Purkinje network was approximated by a partitioned subendocardial surrogate with region-specific isotropic conduction velocities. Whole-heart electrophysiology was simulated using a reaction-eikonal formulation with region-specific ionic models, enabling near real-time computation of cardiac activation and the associated 12-lead electrocardiogram.

**Results:**

Simulated activation sequences reproduced physiologically consistent atrial and ventricular depolarisation patterns, including right atrial breakthrough along the crista terminalis, Bachmann’s bundle-mediated interatrial conduction, dual-pathway atrioventricular nodal activation, and near-synchronous ventricular depolarisation initiated at the fascicle-myocardial junctions. The simulated 12-lead electrocardiogram (ECG) demonstrated close qualitative agreement with the measured recording, with particularly strong agreement in QRS morphology and overall correspondence in P-wave morphology.

**Discussion:**

This framework provides an anatomy-guided and computationally tractable platform for investigating conduction system physiology, bundle branch block phenotypes, and conduction system pacing strategies.

## 1 Introduction

Computational models of cardiac function are central to modern cardiovascular research. By integrating heterogeneous experimental and clinical data into a unified mechanistic framework, they enable multiscale insight, linking ion channel dynamics to emergent phenomena such as wavefront propagation at tissue and organ levels. These models support hypothesis testing *in silico* through controlled, reproducible experiments that allow systematic parameter variation and isolation of causal mechanisms, thereby reducing reliance on animal and invasive human studies (1). Recent advances seek to move beyond generic mechanistic models toward patient-specific frameworks that reconstruct individual anatomy from imaging data and calibrate electrophysiological properties using subject-specific measurements (2, 3). Such patient-specific models, referred to as cardiac digital twins (4), are used to inform risk stratification (5), guide therapy selection, and support treatment planning (6). These applications rely on the assumption that simulated virtual and real physical space are closely intertwined. Yet, a key limitation inevitably leading to discrepancies between simulated and real physiology persists: the inadequate representation of whole-heart electrophysiology, primarily due to the omission or oversimplification of the cardiac conduction system (7, 8).

Cardiac organ-scale function critically depends on coordinated atrioventricular electrical activity mediated by the CCS. Despite its vital role, this system is frequently neglected in computational electrophysiology models. Instead, simulations commonly consider anatomical domains in isolation such as the left atrium (9), atria (10–13), the left ventricle (14) or ventricles (2, 3). Only a limited number of whole-heart models have been reported. Most of these rely on *ad hoc* phenomenological coupling schemes that reproduce selected spatio-temporal activation patterns but do not explicitly represent the underlying anatomy and physiology of the cardiac conduction system (15–17). A notable exception is the model presented in (2). These simplifications limit physiological fidelity and application scope as the cardiac conduction system—comprising sinoatrial node (SAN), atrioventricular node (AVN) and His-purkinje system (HPS)—is always actively involved under both healthy and diseased conditions. It orchestrates normal rhythm generation by its pacemaker function and mediates atrioventricular delay and rapid ventricular activation essential for efficient mechanical performance. It is also directly implicated in the majority of conduction disorders diagnosed based on ECG features, and contributes to arrhythmia initiation and maintenance (18, 19). The persistent omission of the CCS in modelling studies is not due to a lack of recognition of its importance. Rather, it reflects the substantial challenges: embedding its fine-scale, discrete structures within anatomically realistic whole-heart geometries; inferring its structure and calibrating its electrophysiological properties to match limited sparse organ-scale observations such as the ECG (20), while maintaining computational tractability (2). Overcoming these challenges is essential to achieve physiologically comprehensive models, enable the investigation of mechanisms at the whole-heart scale, and facilitate more direct comparison with clinical observations, which inherently reflect the integrated electrical activity of the entire heart.

Mechanistically complete and electrophysiologically accurate predictions require computational models that explicitly represent the full CCS: the SAN and its exit sites initiate right atrium (RA) activation, shaping the early P-wave, while interatrial pathways determine left atrium (LA) activation, reflected in the terminal P-wave. The AVN, with its compact node accessible through slow and fast entrance pathways, regulates atrioventricular delay and chamber synchronization in healthy conditions, but provides also a pathological substrate for tachyarrhythmias such as atrioventricular nodal reentry tachycardia (AVNRT). Ventricular activation is mediated by the fast-conducting HPS—including the His bundle, bundle branches, fascicles, and terminal Purkinje network—which rapidly distributes impulses across the ventricular subendocardium to ensure fast ventricular activation and synchronous contraction. Incorporating an anatomically detailed and physiologically calibrated CCS enhances whole-heart models by providing a mechanistic basis for simulating activation dynamics and depolarisation-related ECG morphology across healthy and diseased conditions, enabling the investigation of arrhythmia mechanisms and the evaluation of therapeutic interventions such as conduction system pacing (CSP) (21).

In this study, we present a comprehensive holistic human whole-heart model incorporating an anatomy-guided, mechanistically detailed representation of the CCS. Our model comprises anatomically accurate representations of the SAN, discrete atrial conduction pathways, an AVN coupled to a micro-anatomically informed His bundle, connected to bundle branches and a distal Purkinje network. Topology and spatial arrangement of the entire conduction axis are informed by anatomical landmarks derived from routine clinical images. Electrophysiology (EP) is represented by a fast physics-informed eikonal model (22) to enable simulation of cardiac depolarisation and the associated ECG with near real-time performance. We show that the anatomy-guided CCS design is consistent with measured P-wave and QRS morphology, confirming the physiological consistency of the modelling framework.

## 2 Materials and methods

### 2.1 Anatomical modelling

A subject-specific anatomical model of the heart and torso was constructed from magnetic resonance imaging of a 45-year-old male subject (ethics approval: Medical University of Graz, EKNr: 24–126 ex 11/12). The segmented geometry provided a high-resolution representation of the atrial and ventricular chambers, with an approximate mesh resolution of 1000 μm, with local refinement to approximately 250 μm within the volumetric nodal regions, embedded within the surrounding torso volume.

Ventricular myocardial fibre architecture was assigned using established rule-based methodologies (23). Fibre orientations were prescribed to rotate linearly from +60${}^{\circ }$ at the endocardium to −60${}^{\circ }$ at the epicardium, while sheet and sheet-normal orientations varied from −65${}^{\circ }$ to 25${}^{\circ }$, consistent with experimental observations of transmural myocardial organisation (23). Atrial fibre architecture was computed using the rule-based framework described in (24), incorporating subsequent methodological refinements that improve robustness and anatomical fidelity (25).

A four-chamber heart mesh served as the anatomical substrate, into which individual CCS and interatrial conduction (IAC) components were constructed and integrated sequentially, as described in the following subsections. All anatomical modelling steps were performed using *NumeriCor Studio* (NumeriCor GmbH, Graz, Austria).

The CCS components were not directly segmented from the subject-specific images. Instead, they were manually reconstructed in *NumeriCor Studio* using anatomical landmarks identifiable in the heart mesh together with geometric descriptions reported in the literature. The SAN, AVN, His bundle, and bundle branches were informed by anatomical and histological studies, whereas the primary Purkinje network (PN) layout was guided by micro-CT data and papillary muscle landmarks. Thus, the heart–torso geometry, chamber anatomy, papillary muscle locations, universal ventricular coordinates (UVC)-based regions, and electrode positions were subject-specific, whereas the CCS anatomy was literature-guided rather than directly imaged.

#### 2.1.1 Sinoatrial node

The SAN is a three-dimensional, markedly heterogeneous tissue composed of pacemaker cells, atrial myocytes, adipocytes, and fibroblasts, each contributing to its complex architecture and electrical behaviour (26–29). Anatomically, it is located at the junction of the superior vena cava (SVC) and extends along the crista terminalis toward the inferior vena cava (30, 31). Although the SAN head normally lies closer to the epicardium and the tail extends toward the subendocardial region (32), a simplified representation was adopted, in which the entire structure was positioned at a uniform depth of approximately 2 mm from the epicardial surface to reduce modelling complexity (33) (Figure 1). The SAN dimensions were defined as 12 mm in length, 3 mm in width, and 2 mm in thickness, derived from averaged values reported across anatomical studies (34–36).

**Figure 1:**
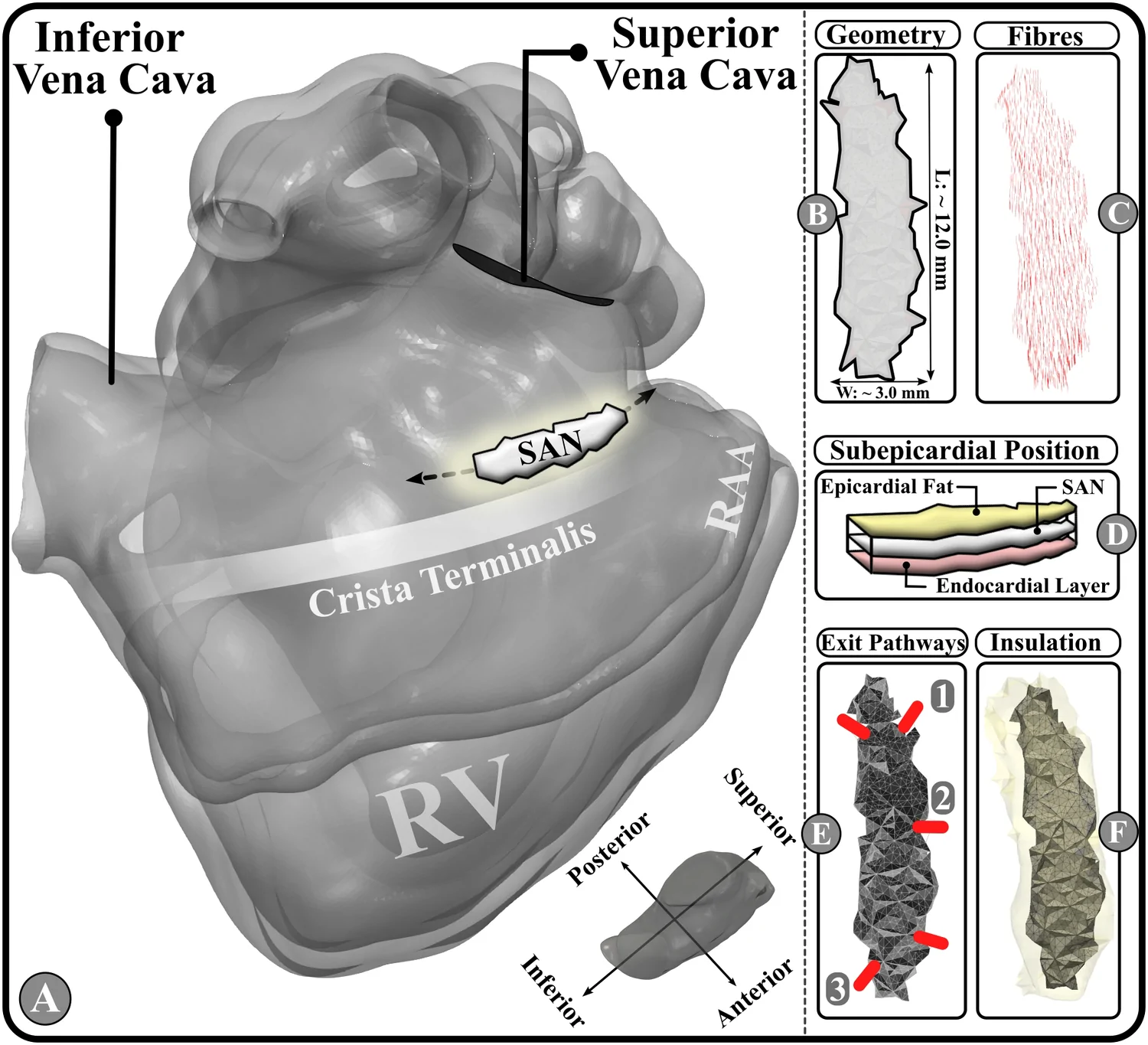
Anatomical representation of the sinoatrial node (SAN) within the human right atrium. **(A)** Location of the SAN at the junction of the superior vena cava, extending along the crista terminalis. **(B)** Three-dimensional geometry of the SAN. **(C)** Unidirectional fibre orientation aligned with the long axis of the SAN. **(D)** Subepicardial positioning of the SAN relative to the atrial wall. **(E)** Distribution of five anatomically reported SAN exit pathways in the human heart (highlighted in red); three of these pathways were incorporated in the present model as 1D (red) cables to provide electrical coupling between the SAN and the surrounding atrial myocardium; the remaining two locations are shown only for anatomical context. **(F)** Electrical insulation of the nodal tissue from adjacent working myocardium.

The SAN was modelled as a structure embedded in RA tissue and electrically insulated by a surrounding layer of fibrous connective tissue, which limits electrotonic coupling with the surrounding atrial myocardium and preserves stable pacemaking (30, 37). Its fibres were oriented unidirectionally along the longitudinal axis, in agreement with previous measurements (35, 36). Within this configuration, electrical interaction with the atria was permitted only through a limited number of defined exit pathways, through which impulses propagate from the SAN into the atrial myocardium (26, 34, 38). This approach was adopted to preserve the intrinsic behaviour of the node while reproducing an atrial activation sequence consistent with measured imaging and mapping data (33, 36, 39, 40).

The SAN exit pathways implemented in the model were represented as 1 mm length 1D cables traversing the insulating layer to form discrete conductive bridges between the SAN and neighbouring atrial myocardium. Reported anatomical studies describe between two and five SAN exit pathways, categorised according to their spatial orientation as lateral, medial, superior, and anterior pathways (32, 40). In Figure 1, five anatomically reported SAN exit pathways locations are shown for anatomical context. In the present model, three of these pathways were electrically implemented to provide controlled atrial coupling: one lateral cranial pathway near the superior aspect of the node (1), one medial caudal pathway directed toward the lateral region (2), and one inferior pathway located at the nodal tail (3). The remaining two reported SAN exit pathways locations were not electrically coupled to the atrial myocardium in this model.

Although a characteristic “block zone” has been reported surrounding the SAN, introducing anatomical and physiological barriers that restrict conduction and activation along preferred pathways (30, 35), this feature was omitted. The anatomical site of excitation within the SAN can vary depending on the dominant pacemaker region (30, 41), with impulses originating in the central body, head, or tail. Within the model, excitation propagated gradually through the SAN before reaching the nearest SAN exit pathways, which subsequently mediated depolarisation of the adjacent atrial myocardium and thereby initiated the P-wave on the surface ECG (26, 32, 33, 37, 42–44). Sinus activation was prescribed in the central region of the SAN, consistent with literature describing this region as a leading pacemaker location (45).

#### 2.1.2 Interatrial conduction pathways

IAC was represented by incorporating specialised pathways that facilitate the rapid transmission of excitation from the RA to the LA (46, 47). Although the atria do not possess insulated fibres analogous to those of the specialised conduction system, contemporary anatomical studies have demonstrated that certain regions contain cardiomyocytes organised into parallel bundles capable of preferential and directionally aligned conduction (48, 49). The Bachmann’s bundle (BB), recognised as the primary interatrial connection, was therefore modelled explicitly. Anatomically, BB consists of parallel-aligned myocardial strands forming a muscular bridge that extends subepicardially across the interatrial groove and remains distinguishable from the surrounding atrial tissue (50–52). Unlike the SAN, this structure is not insulated by a fibrous sheath and is instead composed entirely of atrial fibres arranged to support rapid, directed conduction (52).

To reproduce these characteristics, BB was represented by 10 parallel one-dimensional cables with identical conduction velocity, originating between the SVC and the right atrial appendage (RAA) and terminating at spatially distributed insertion sites around the left atrial appendage (LAA) (Figure 2). This multi-cable representation was used to approximate the broad anatomical structure of BB and to distribute BB-mediated activation over a larger left atrial region, avoiding a point-like breakthrough produced by a single cable.

**Figure 2:**
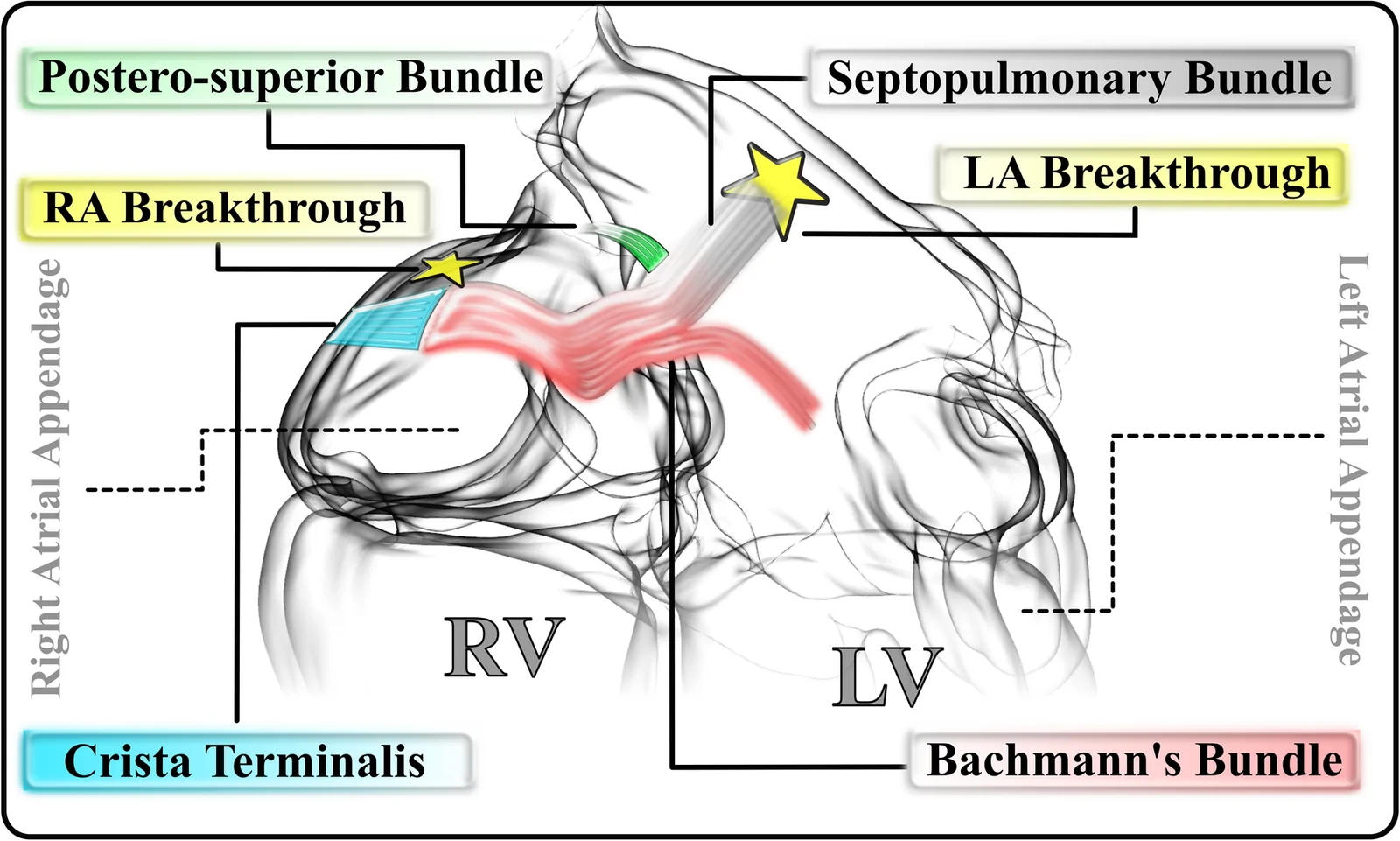
Anatomical representation of the interatrial conduction pathways within the human atria. Parallel one-dimensional cables depict the organised strands of Bachmann’s bundle (red) coursing across the interatrial septum to reach the left atrial appendage. The posterosuperior bundle (green) originates posterior to the superior vena cava and converges with Bachmann’s bundle, providing an alternative posterior IAC route. The septopulmonary bundle (grey), representing the left atrial continuation of Bachmann’s bundle, extends across the superior dome of the left atrium.

In addition to BB, IAC also traversed posterior routes located within the Waterston groove (43). These pathways, typically described as posterosuperior bundles near the RA–SVC junction, were implemented again as one-dimensional cables (Figure 2). The posterosuperior bundle (PSB) was further connected to the posterior margin of the BB across the interatrial groove, forming an integrated network capable of supporting alternative IAC routes when anterior pathways are less dominant (53).

An additional pathway considered was the rim of the fossa ovalis (FO) and septopulmonary bundle (SPB). The existence of the SPB remains debated, with some studies reporting its presence (53, 54), while others do not (55, 56). In the present model, this bundle was included and extended from the interatrial groove toward the roof of the LA, providing a potential anatomical substrate for left atrial breakthrough in this region (53, 54).

#### 2.1.3 Atrioventricular node

The AVN was modelled as the central structure governing atrioventricular synchrony, reflecting its physiological role in imposing the conduction delay that regulates impulse transmission from the atria to the ventricles and contributes to the PR interval (57, 58). The node (Figure 3) was positioned within the triangle of Koch (ToK), located on the RA surface of the interatrial septum (IAS) and forming the apex of the triangular region bounded by the ostium of the coronary sinus, the tendon of Todaro (ToT), and the hinge line of the septal leaflet of the tricuspid valve (TV) (59, 60). The central component of the AVN, the compact node (CN), exhibits variable morphology in anatomical studies, including triangular, elliptical, and stellate forms (61–63). For modelling purposes, a disk-like structure was implemented at the apex of the ToK, with a diameter of 5.0 mm and a thickness of 2 mm, based on averaged anatomical measurements (64). The dual-pathway organisation of the AVN was reproduced by incorporating distinct fast pathway (FP) and slow pathway (SP) components. The FP was connected from an anterior trajectory across the IAS to represent the transitional region leading into the CN (65, 66), whereas the SP originated from a posterior region as a continuation of the terminal crest and entered the CN via the TV isthmus (67). Although both pathways are electrically active under physiological conditions, the fast and slow terminology reflects their overall conduction time rather than necessarily their local conduction velocity. The FP can exhibit a slightly lower local conduction velocity than the SP; however, because it follows a shorter anatomical route to the CN and subsequently the penetrating His-bundle (PHB), activation can arrive earlier through the FP than through the SP (57, 68). Together, these pathways formed a dual-input system converging at the AVN.

**Figure 3:**
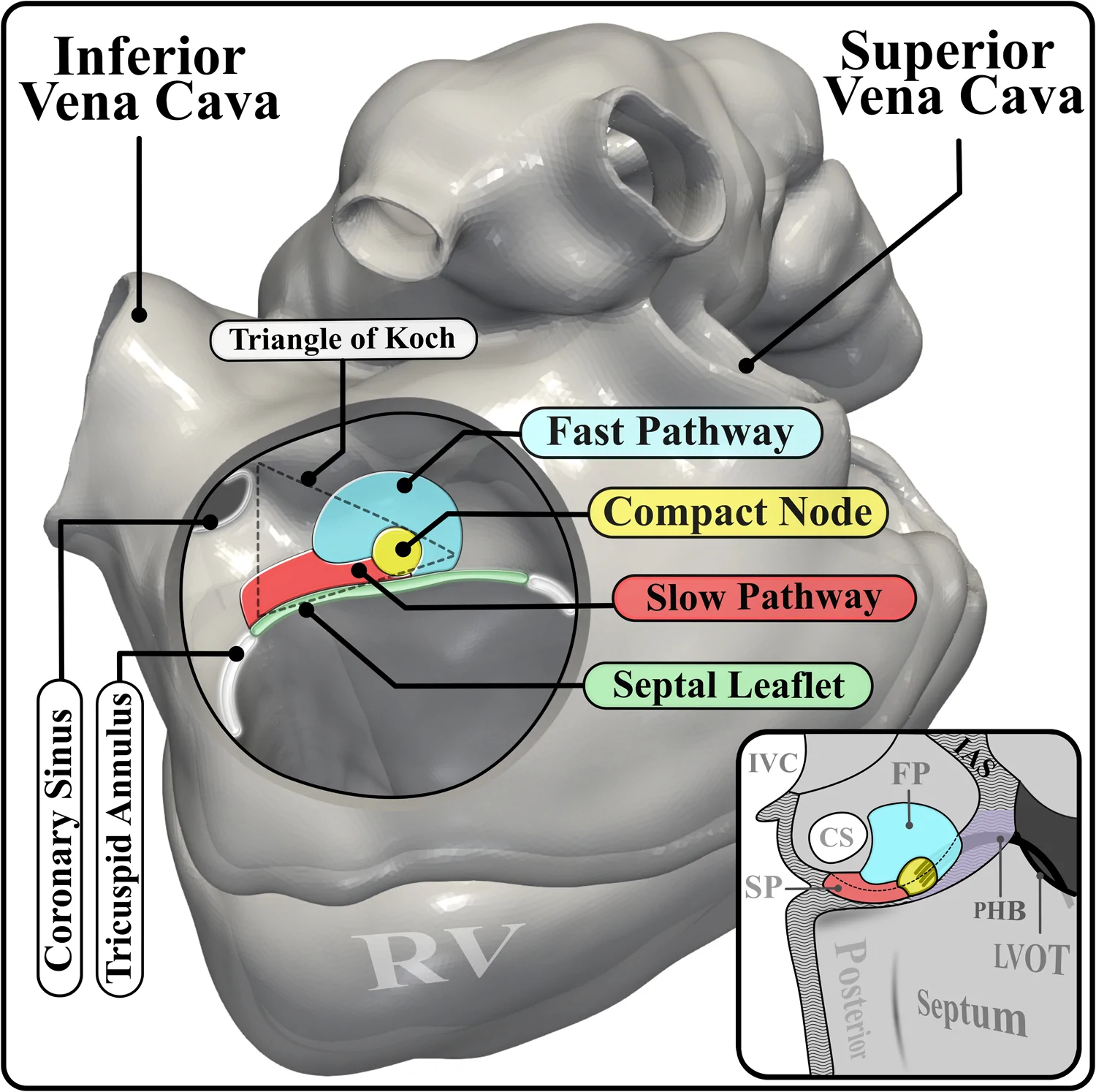
Anatomical representation of the atrioventricular node situated within the triangle of Koch, adjacent to the septal leaflet of the tricuspid valve. The compact node (yellow) is shown at the nodal core, with the fast pathway (blue) extending from the anterior interatrial septum and representing the transitional cell region, and the slow pathway (red) approaching the nodal complex from an inferior and posterior direction.

#### 2.1.4 His-bundle

The His-bundle (HB) represents the distal continuation of the AVN, beginning as a penetrating segment that directly extends from the compact node, traverses the membranous septum in an anterior and inferior direction, and becomes encased within the central fibrous body (CFB) (69–71). Anatomically, the HB was considered to comprise three segments: the PHB that passed through the fibrous membranous septum, a variable non-branching mid-tract that coursed along the crest of the muscular ventricular septum for a further 1–3 mm, and a distal branching segment adjacent to the aortic ring where bifurcation into the left and right bundle branches occurred (71–75). The PHB entry into the CFB on the atrial side of the septal tricuspid hinge was noted and used to inform the proximal geometry of the modelled trunk (76, 77). The HB was implemented as four parallel cable elements emerging from the AVN (Figure 4) to form the principal fascicular trunks of the left bundle branch (LBB) and right bundle branch (RBB), and these cables were electrically insulated from the adjacent working myocardium in accordance with classical observations (78). No lateral connections were present along the trunk; electrical coupling between the cable elements was restricted to the PHB region. Finally, the more common left-sided alignment of the HB was adopted in the modelled geometry to match typical anatomical orientation (Figure 5) (79).

**Figure 4:**
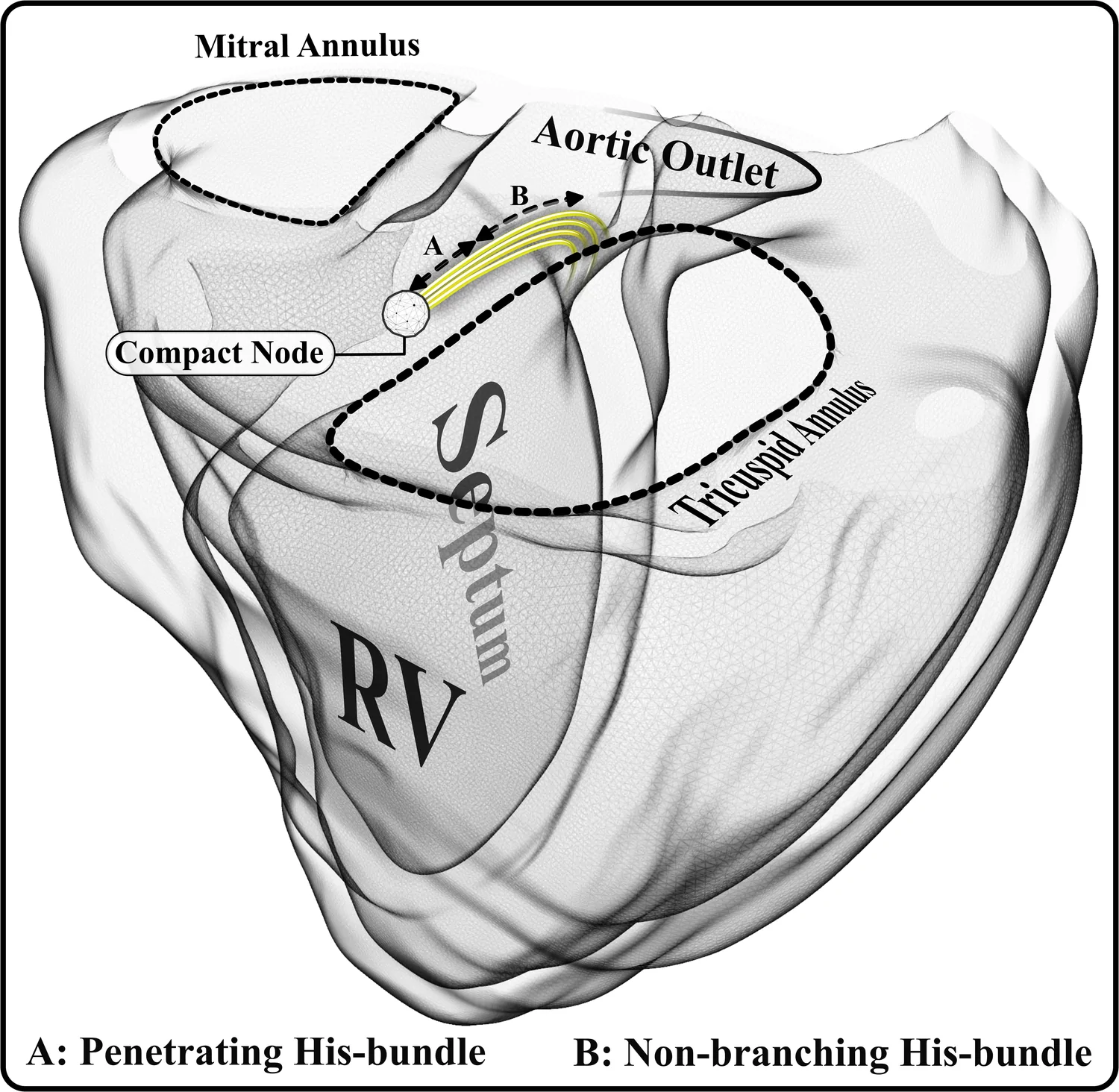
Anatomical representation of the His bundle within the human heart model. The penetrating His-bundle is depicted as the distal continuation of the AVN, emerging from the compact node and traversing the membranous septum. Distal to the penetrating His-bundle, a short non-branching His segment courses along the crest of the muscular ventricular septum for approximately 1–3 mm before giving rise to the bundle branches.

**Figure 5:**
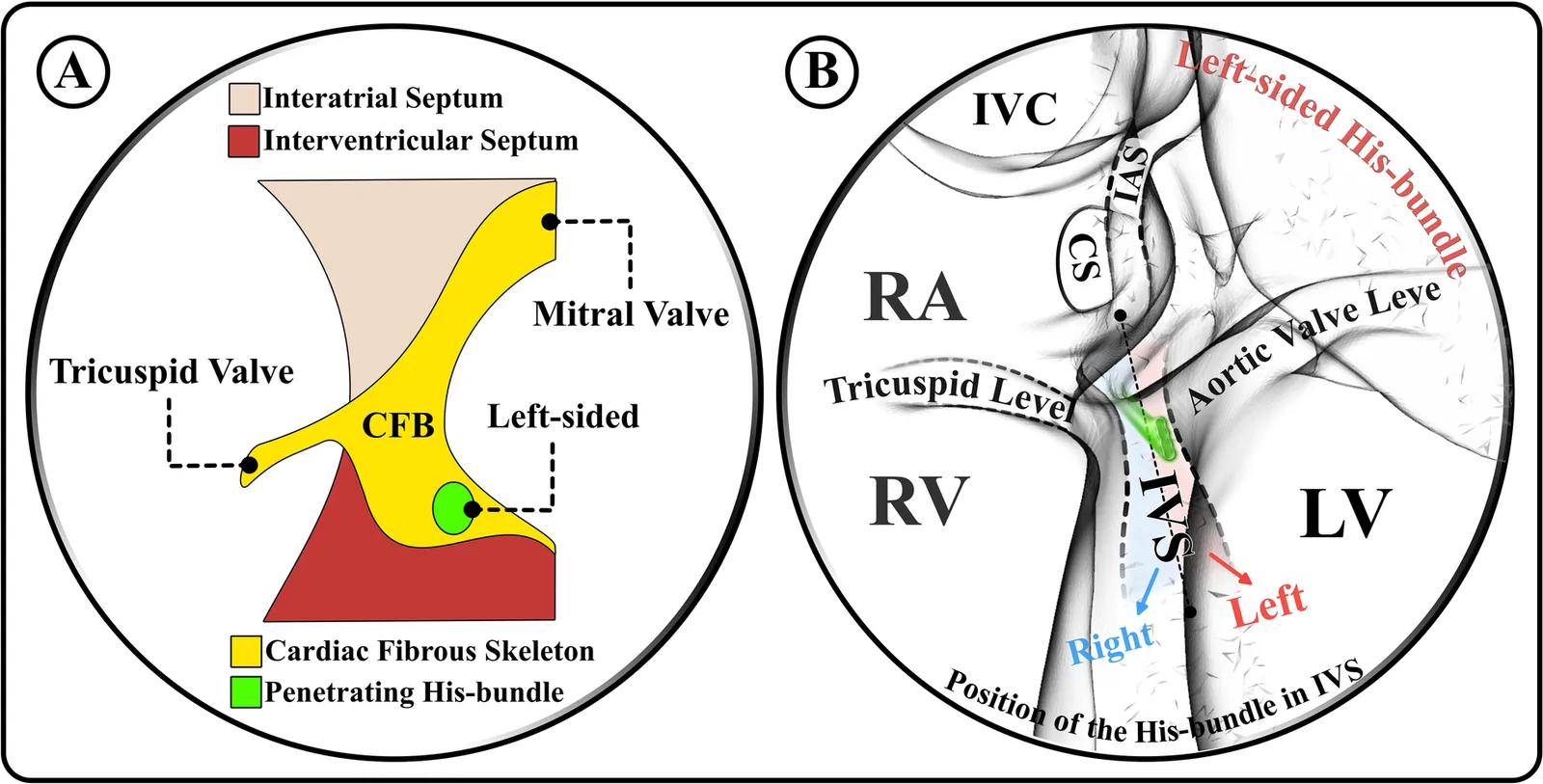
Left-sided position and intraseptal course of the penetrating His bundle (green) within the interventricular septum (IVS). **(A)** The PHB is positioned on the left side of the septum below the level of the tricuspid valve. **(B)** Three-dimensional view showing the non-branching PHB coursing intraseptally through the IVS before aligning along the left side of the septal wall and bifurcating into the LBB and RBB.

#### 2.1.5 Bundle branches

The LBB was modelled to reflect its anatomical origin and early intraventricular organisation, arising inferior to the membranous septum at the junction between the right and non-coronary aortic cusps, where fibres emerge from the distal HB. Anatomical studies describe the proximal LBB as a compact, ribbon-like structure positioned immediately beneath the subaortic endocardium within the angle formed by the right and non-coronary cusps (72, 79, 80). To reproduce this morphology within the computational framework, the proximal LBB was represented by multiple closely spaced 1D cables arranged in parallel and maintained electrically uncoupled from the surrounding myocardium (Figure 6). Although implemented as distinct cables, they were arranged in close apposition in the proximal segment to mimic an undivided ribbon-like bundle that descends inferiorly and slightly anteriorly prior to their distal divergence into anatomically defined fascicles (72, 79, 81). A trifascicular configuration was then implemented, in accordance with widely accepted structural classifications (82, 83). The posterior fascicle branched first and swept posteriorly toward its termination near the base of the posterior papillary muscle; the septal fascicle proceeded distally in an apicobasal trajectory and terminated at approximately the mid-apicobasal level; and the anterior fascicle arose from the anterior border of the trunk, following an oblique course beneath the left ventricle (LV) outflow tract toward the base of the anterior papillary muscle (80, 83).

**Figure 6:**
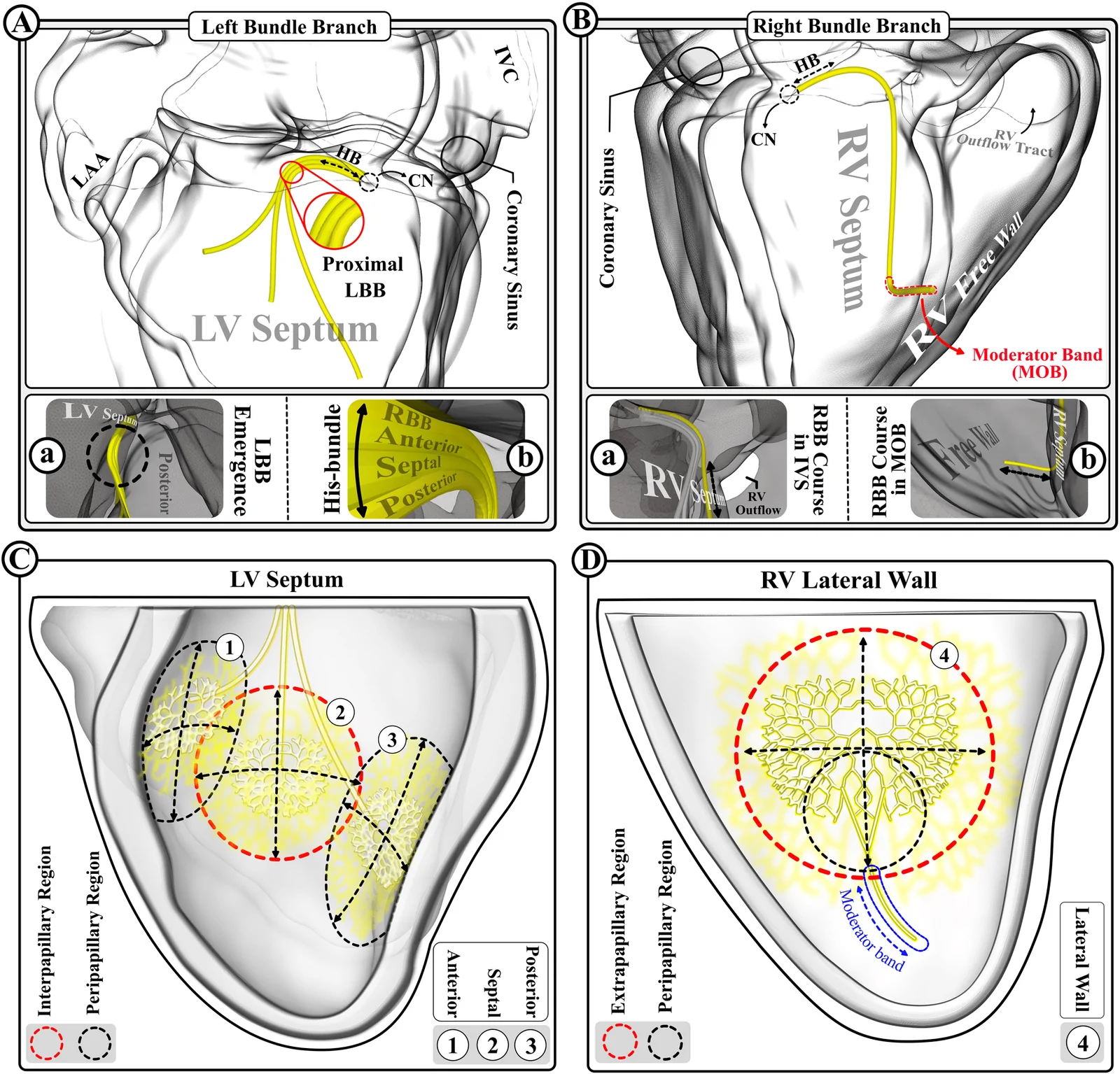
Anatomical representation of the ventricular conduction system, including the left bundle branch (LBB), right bundle branch (RBB), and associated Purkinje networks (PN). **(A)** The proximal LBB is depicted as a compact, band-like structure emerging from the HB and dividing distally into its major fascicular trunks; inset **(a)** illustrates its endocardial emergence, and inset **(b)** indicates the origin of the ventricular conduction axis. **(B)** The RBB is shown as a single trunk coursing within the interventricular septum **(a)** and traversing the moderator band distally **(b)** before giving rise to the right ventricular PN. **(C)** Primary left ventricular PN at the fascicle–myocardial junction; dashed black regions denote prepapillary territories, and the red dashed line marks the septal interpapillary region. **(D)** Primary right ventricular PN at the fascicle–myocardial junction; dashed black regions indicate the anterior peripapillary area, and the red dashed line highlights the extrapapillary region along the lateral wall. In **(C)** and **(D)**, the solid yellow lines represent the explicitly modelled primary Purkinje-network cables, while the black dashed arrows indicate the manually adjusted spatial extent of the primary Purkinje network during ECG calibration.

The RBB trunk was represented as a separate fascicle positioned adjacent to the most anterior fascicle of the LBB, consistent with their partially shared origin (80). The trunk was assigned a short intramuscular course through the IVS before emerging onto the subendocardial surface of the right ventricle (RV) beneath the base of the medial papillary muscle (72, 79, 84–86). Similar to the LBB, the RBB was modelled without electrical coupling to the surrounding myocardium to preserve its distinct conduction properties (72). The RBB coursed through the RV (Figure 6B) toward the anterolateral papillary muscle, consistent with anatomical observations that the RBB traverses the RV via the moderator band before giving rise to a distributed PN spanning the RV free wall (74, 79, 84, 87).

#### 2.1.6 Purkinje network

The terminal component of the CCS, comprising the cardiac PN, was modelled to rapidly and synchronously distribute electrical activation throughout the ventricular myocardium (88). Anatomical studies have shown that, as the bundle branches extend toward the papillary muscles, the Purkinje fibers (PFs) undergoes progressive branching to form a dense subendocardial network (89). Consistent with these observations, each fascicular trunk gave rise to a dense network of PFs represented as interconnected one-dimensional cables. In the LV, the anterior and posterior primary networks were localised near the corresponding papillary muscle bases, while the septal network spanned the interpapillary septal region between them. In the RV, the primary PN was implemented along the lateral wall around the anterior papillary muscle and moderator-band root. These terminal locations were fixed by anatomical landmarks, whereas the spatial extent of the primary PN was adjusted manually during ECG calibration. Proximally, the PFs are electrically insulated from the surrounding working myocardium by a connective tissue sheath, such that electrical continuity occurs only at their distal terminations through specialised coupling at the Purkinje-ventricular junction (PVJ) (90). Conduction across these junctions is inherently discontinuous, with measurable activation delays over short spatial scales, as reported in experimental and computational studies (91, 92). Coupling between the PFs and the ventricular mesh was therefore restricted to terminal sites, with a fixed delay of 3 ms imposed at each PVJ to reproduce activation transfer with a delay consistent with reported PVJ measurements (91). Moreover, based on human-specific observations indicating that the majority of PFs are located on the endocardial surface (88), intramural components of the PN were not included.

#### 2.1.7 Ventricular sub-endocardial regions

The subject-specific regional ramification topology of the distal PN is not accessible from available imaging data; a surrogate representation was therefore adopted. The subendocardium of each ventricle was partitioned into septal, anterior, and posterior regions using the UVC framework (93) (Figure 7), with regionally differentiated isotropic conduction velocities assigned to each zone to govern the spread of activation following endocardial breakthrough at the fascicle–myocardial junctions. The corresponding coordinate ranges are summarised in Table 1.

**Figure 7:**
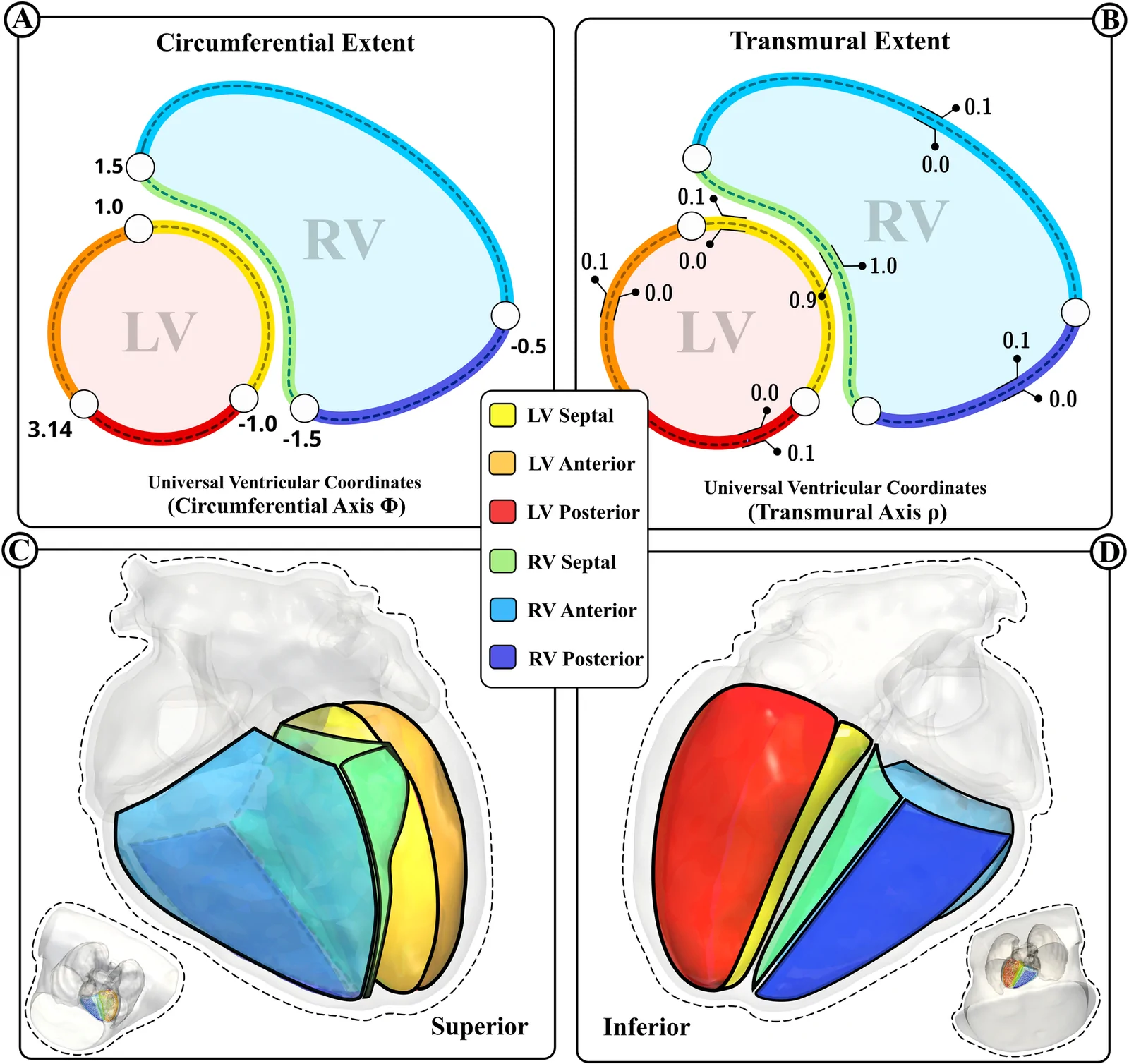
Subendocardial region definitions in the left and right ventricles using Universal Ventricular Coordinates (UVC) (93). **(A)** Circumferential extent of the anterior, septal, and posterior subendocardial regions in the LV and RV defined along the UVC ϕ-axis. **(B)** Transmural extent of the same regions along the UVC ρ-axis, illustrating consistent subendocardial layer delineation across both ventricles. **(C)** Superior and **(D)** inferior views of the partitioned subendocardial regions, demonstrating their three-dimensional spatial distribution.

**Table 1:** Universal ventricular coordinates defining the partitioned subendocardial regions of the ventricles.

Regions	ϕ-axis	ρ-axis	ν-axis
LV septal	−1.00 to 1.00	0.0–0.1	−1.0
LV anterior	1.00–3.14	0.0–0.1	−1.0
LV posterior	−3.14 to −1.00	0.0–0.1	−1.0
RV septal	−1.50 to 1.50	0.9–1.0	1.0
RV anterior	−0.50 to 1.50	0.0–0.1	1.0
RV posterior	−1.50 to −0.50	0.0–0.1	1.0

In the UVC convention, ρ=0 denotes the endocardium, while the RV septal surface corresponds to the LV epicardium and is therefore defined by ρ=1.

This representation was chosen intentionally to distinguish between explicitly modelled anatomical structures and unresolved distal components of the ventricular conduction system. The proximal bundle branches, fascicles, and primary Purkinje-network extensions were defined from anatomical landmarks, including the papillary muscle regions and septal fascicular insertion sites, because these structures determine the earliest ventricular breakthrough locations. In contrast, the subject-specific topology of the distal Purkinje ramifications cannot be reconstructed from available clinical imaging data. These unresolved ramifications were therefore represented by regional fast subendocardial layers, serving as an established surrogate for rapid endocardial activation. This approach avoided speculative reconstruction of individual Purkinje branches while preserving the dominant effect of early endocardial excitation on QRS morphology.

### 2.2 Electrophysiological simulations

Whole-heart electrophysiological activity was simulated using a reaction–eikonal formulation, which enables computationally efficient representation of activation timing while preserving wavefront propagation at conduction velocities consistent with more expensive high-resolution reaction–diffusion models (94). Each anatomical component of the CCS and working myocardium was assigned a region-specific ionic model, conduction velocity, anisotropy ratio, and numerical representation, as summarised in Table 2. The atrial and ventricular myocardium was represented as an orthotropic conductive medium, with conduction velocities prescribed in a 2:1:1 ratio along the fibre, sheet, and sheet-normal directions, respectively. Atrial myocyte dynamics were governed by the (95) model, ventricular myocyte dynamics by the (106) model, and specialised conduction system electrophysiology by the (105) Purkinje cell model. Before whole-heart simulation, the single-cell ionic models were pre-paced to steady state at a basic cycle length of 600 ms for 10 beats (96). The resulting steady-state variables were used as initial conditions for the whole-heart model. A single sinus beat was then simulated, and the reported ECG corresponds to this first whole-heart beat after pre-pacing.

**Table 2:** Electrophysiological parameters assigned to all cardiac regions and conduction system components.

Component/Region	Ionic model	CV (m/s)	Reported (m/s)	Anisotropy	Representation
Sinoatrial node					
Leading Pacemaker	Fabbri et al.	0.1	0.09–0.10 (35)	1:1:1	Volumetric mesh
SAN exit pathways	Fabbri et al.	0.1	0.03–0.12 (35)	–	1D cable
Atrial myocardium					
CT	Courtemanche et al.	0.7	0.80–0.90 (99)	2:1:1	Volumetric mesh
RA	Courtemanche et al.	0.7	0.60–1.00 (100)	2:1:1	Volumetric mesh
RAA	Courtemanche et al.	0.8	0.60–1.00 (100)	2:1:1	Volumetric mesh
LA	Courtemanche et al.	1.0	0.60–1.20 (101)	2:1:1	Volumetric mesh
LAA	Courtemanche et al.	0.8	NA	2:1:1	Volumetric mesh
Interatrial conduction					
BB	Courtemanche et al.	2.0	0.60–1.30 (102)	–	1D cable
PSB	Courtemanche et al.	1.0	NA	–	1D cable
SPB	Courtemanche et al.	2.0	NA	–	1D cable
FO Rim	Courtemanche et al.	1.0	NA	1:1:1	Volumetric mesh
Atrioventricular node					
FP	Courtemanche et al.	0.04	0.04–0.05 (57)	1:1:1	Volumetric mesh
SP	Courtemanche et al.	0.03	0.03–0.04 (57)	1:1:1	Volumetric mesh
CN	Courtemanche et al.	0.02	0.02–0.05 (57)	1:1:1	Volumetric mesh
His bundle					
PHB	Stewart et al.	1.5	1.30–1.70 (63)	–	1D cable
Left bundle branch					
Anterior fascicle	Stewart et al.	1.9	NA	–	1D cable
Septal fascicle	Stewart et al.	3.0	NA	–	1D cable
Posterior fascicle	Stewart et al.	2.3	NA	–	1D cable
Right bundle branch					
RBB fascicle	Stewart et al.	3.0	NA	–	1D cable
Ventricular myocardium					
LV	Ten Tusscher & Panfilov	0.65	0.56–0.95 (103)	2:1:1	Volumetric mesh
RV	Ten Tusscher & Panfilov	0.75	0.81–0.98 (104)	2:1:1	Volumetric mesh
LV Purkinje network${}^{a}$					
Anterior network	Stewart et al.	1.3	NA	–	1D cable
Anterior subendo	Ten Tusscher & Panfilov	1.4	NA	1:1:1	Volumetric mesh
Septal network	Stewart et al.	2.5	NA	–	1D cable
Septal subendo	Ten Tusscher & Panfilov	1.2	NA	1:1:1	Volumetric mesh
Posterior network	Stewart et al.	1.95	NA	–	1D cable
Posterior subendo	Ten Tusscher & Panfilov	1.5	NA	1:1:1	Volumetric mesh
RV Purkinje network${}^{a}$					
Freewall network	Stewart et al.	2.3	NA	–	1D cable
Anterior subendo	Ten Tusscher & Panfilov	2.0	NA	1:1:1	Volumetric mesh
Septal subendo	Ten Tusscher & Panfilov	1.8	NA	1:1:1	Volumetric mesh
Posterior subendo	Ten Tusscher & Panfilov	1.4	NA	1:1:1	Volumetric mesh

CV, conduction velocity. Anisotropy ratios are given along the fibre, sheet, sheet-normal directions; dashes indicate 1D cable elements for which directional anisotropy is not applicable. Reported CVs are included to enable direct comparison with the calibrated values used in the model. Ionic models correspond to Fabbri et al. (38), Courtemanche et al. (95), Stewart et al. (105), and Ten Tusscher and Panfilov (106).

${}^{a}$For each Purkinje network region, two complementary representations are listed. The 1D cable governs rapid conduction to the earliest endocardial breakthrough sites at the fascicle–myocardial junctions. The regional endocardial layer, represented as a volumetric subendocardial mesh with elevated isotropic conduction velocity, serves as a surrogate for the surrounding Purkinje ramification topology, which cannot be reconstructed from available imaging data. All conduction velocities reported here were tuned during ECG calibration and guided by literature-reported values where available, rather than strictly constrained to these ranges.

All simulations were performed using the CARPentry simulation framework (97, 98).

#### 2.2.1 Electrocardiogram

The torso model incorporated precise, image-based ECG electrode positions to compute a standard 12-lead ECG from the simulated cardiac electrical sources using a lead-field formulation (107).

Region-specific conduction velocities listed in Table 2 were calibrated using a staged, manually supervised iterative procedure. The calibration target was agreement between simulated and measured 12-lead ECGs in waveform morphology, duration, and lead polarity, while maintaining physiologically plausible activation sequences. Calibration was performed sequentially. First, atrial and atrial-conduction-system velocities were adjusted against the measured P-wave. These parameters were then fixed while ventricular and His–Purkinje-related velocities were adjusted against the measured QRS complex. The anatomical locations of the principal conduction-system structures and Purkinje terminal regions were kept fixed; however, as described in Section 2.1.6, the spatial extent of the primary Purkinje networks was manually adjusted during QRS calibration. Conduction velocities were guided by values reported in the literature, and parameter changes producing implausible activation sequences were rejected. Calibration was stopped when further parameter adjustments did not provide a meaningful improvement in ECG agreement without compromising the physiological plausibility of the activation sequence.

Simulated ECG traces were processed with a 60 Hz low-pass filter for direct comparison with measured recordings.

For quantitative comparison, measured and simulated ECGs were normalised and analysed within globally defined P-wave and QRS windows. Global P-wave onset and offset, QRS onset and offset, and the isoelectric interval between P-wave offset and QRS onset were identified in both recordings. In addition, lead-specific Pearson correlation coefficients were computed separately for the P-wave and QRS complex to quantify morphology agreement across the 12 leads.

## 3 Results

### 3.1 Atrial activation

Atrial activation was initiated within the SAN, with the earliest excitation originating from the leading pacemaker region located centrally within the node. Following nodal activation, excitation entered the RA through the nearest SAN exit pathway, namely the lateral superior (head) and medial inferior (tail) pathways. This resulted in initial atrial breakthrough along the CT and in the region inferior to the SVC, establishing the early right atrial activation sequence (Figure 8A). From the initial breakthrough site, excitation propagated rapidly along the CT, spreading superiorly toward the SVC and inferiorly toward the inferior vena cava (IVC), while also extending laterally across the right atrial free wall and medially toward the IAS (Figure 8B). The wavefront propagating medially toward the IAS advanced distally to reach the ToK region (Figure 9A) and activated the AVN via the FP (Figure 9B). In parallel, a second wavefront travelling inferiorly along the CT toward the IVC activated the isthmus of the TV (Figure 9B) and subsequently reached the AVN via the SP. These two wavefronts converged at the ToK (Figure 9C), with activation via the SP occurring slightly later than via the FP.

**Figure 8:**
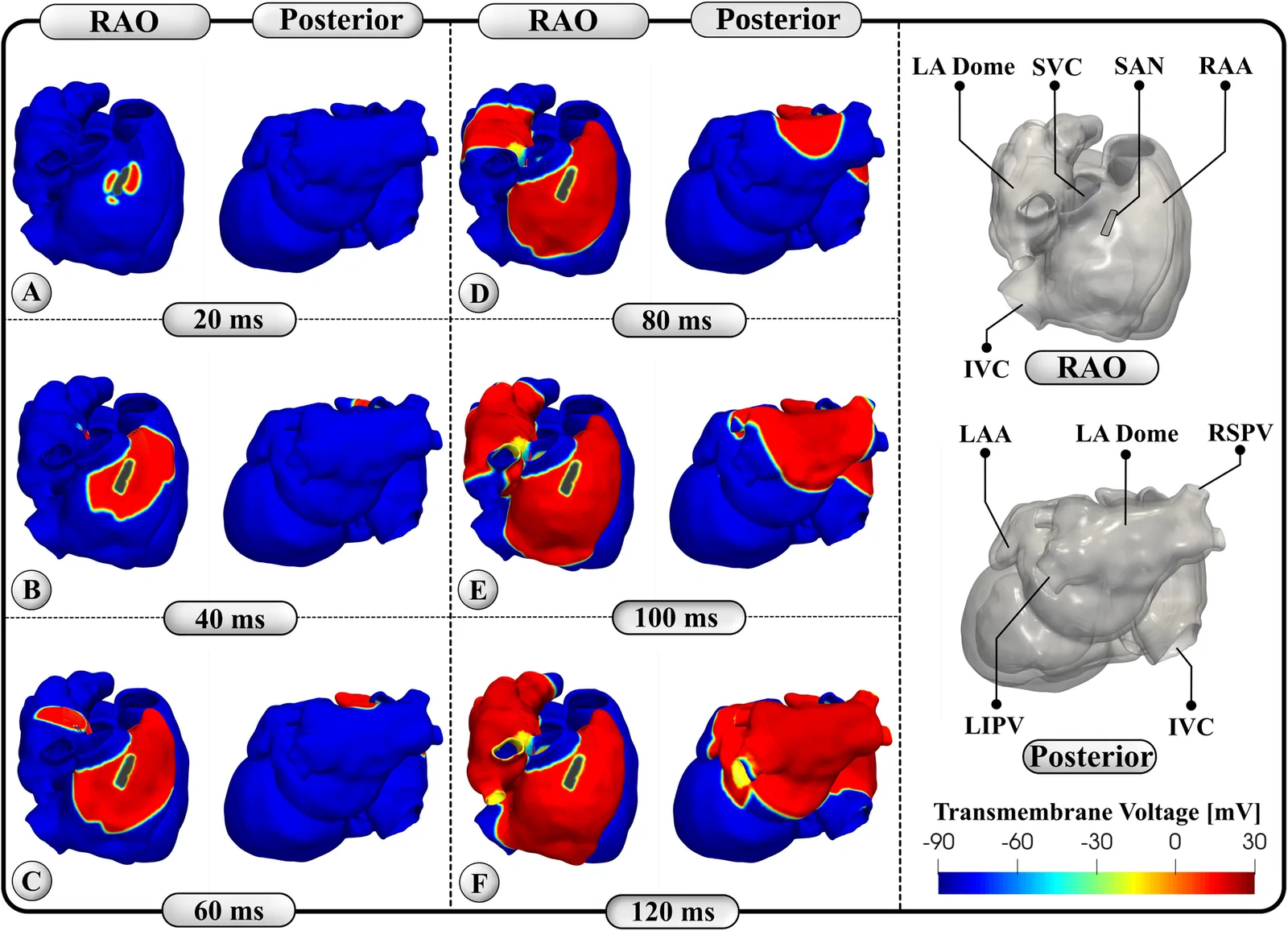
Simulated atrial activation in the human heart model from right anterior oblique (RAO) and posterior views. **(A)** Activation originated within the SAN. **(B)** Right atrial excitation propagated along the CT toward the SVC and IVC. **(C)** IAC was predominantly mediated by the BB, with early left atrial breakthrough at the superior left atrial dome via the SPB continuation of BB. **(D–F)** Subsequent activation of the LAA and completion of atrial depolarisation are shown, including the convergence of wavefronts at the left atrial isthmus. The grey region denotes SAN. Owing to its predominantly subepicardial location and insulation by overlying adipose and connective tissue, transmembrane voltage signals originating from the SAN were not directly observable on the epicardial surface.

**Figure 9:**
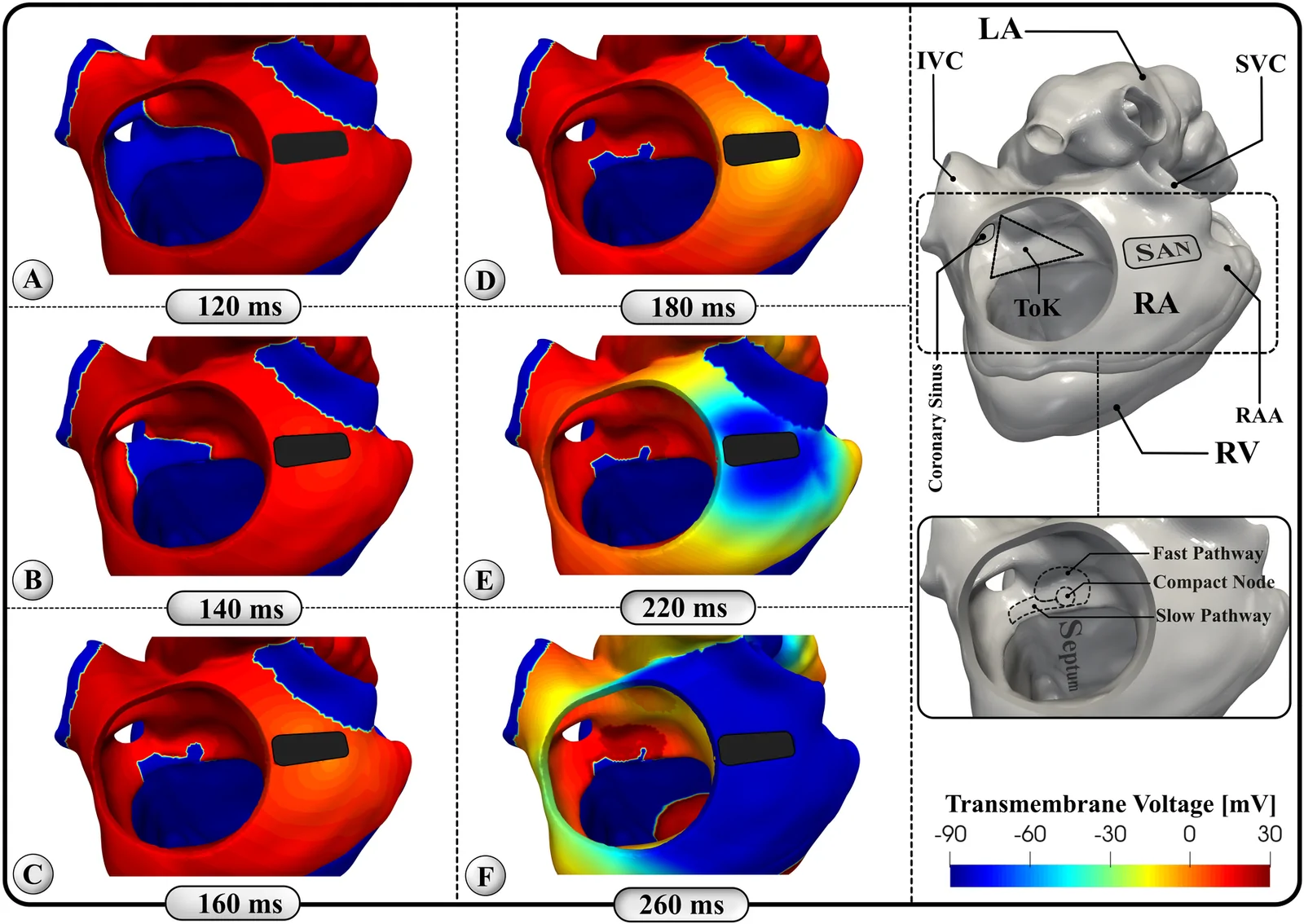
Simulated activation of the AVN in the human heart model. **(A)** Medially directed right atrial wavefront diffusing from the IAS toward the ToK region via the FP. **(B)** A second wavefront approaching the ToK from the inferior right atrium along the CT and across the tricuspid isthmus. **(C)** Collision of the two atrial wavefronts at the ToK, consistent with convergence of fast and slow atrial inputs. **(D–E)** Slow and delayed activation within the AVN. **(F)** Septal ventricular breakthrough reflecting transmission of excitation from the distal AVN to the ventricular conduction system. The grey region denotes the intramural SAN. Owing to its predominantly subepicardial location and insulation by overlying adipose and connective tissue, transmembrane voltage signals originating from the SAN were not directly observable on the epicardial surface.

IAC was predominantly mediated by BB, with activation emerging from the region near the SAN–SVC junction and propagating toward the LA. The earliest LA breakthrough occurred at the atrial roof, where activation was delivered via the SPB as a continuation of BB, resulting in early excitation of the superior portion of the LA dome (Figures 8B,C). As BB advanced toward the vicinity of the LAA, a secondary activation site was observed near the LAA. Given the dominant role of BB in IAC, the PSB contributed minimally under normal sinus rhythm. Ultimately, two wavefronts—one originating from the LA dome and the other from the LAA—converged at the left atrial isthmus, marking completion of atrial activation (Figure 8F).

### 3.2 Ventricular activation

Ventricular activation was initiated by HPS excitation. Following activation of the PHB via the CN (Figure 10C), excitation rapidly bifurcated into the left and right bundle branches. Within the LBB, all three major fascicular trunks—anterior, septal, and posterior—were activated nearly simultaneously, in parallel with activation of the RBB (Figure 10D).

**Figure 10:**
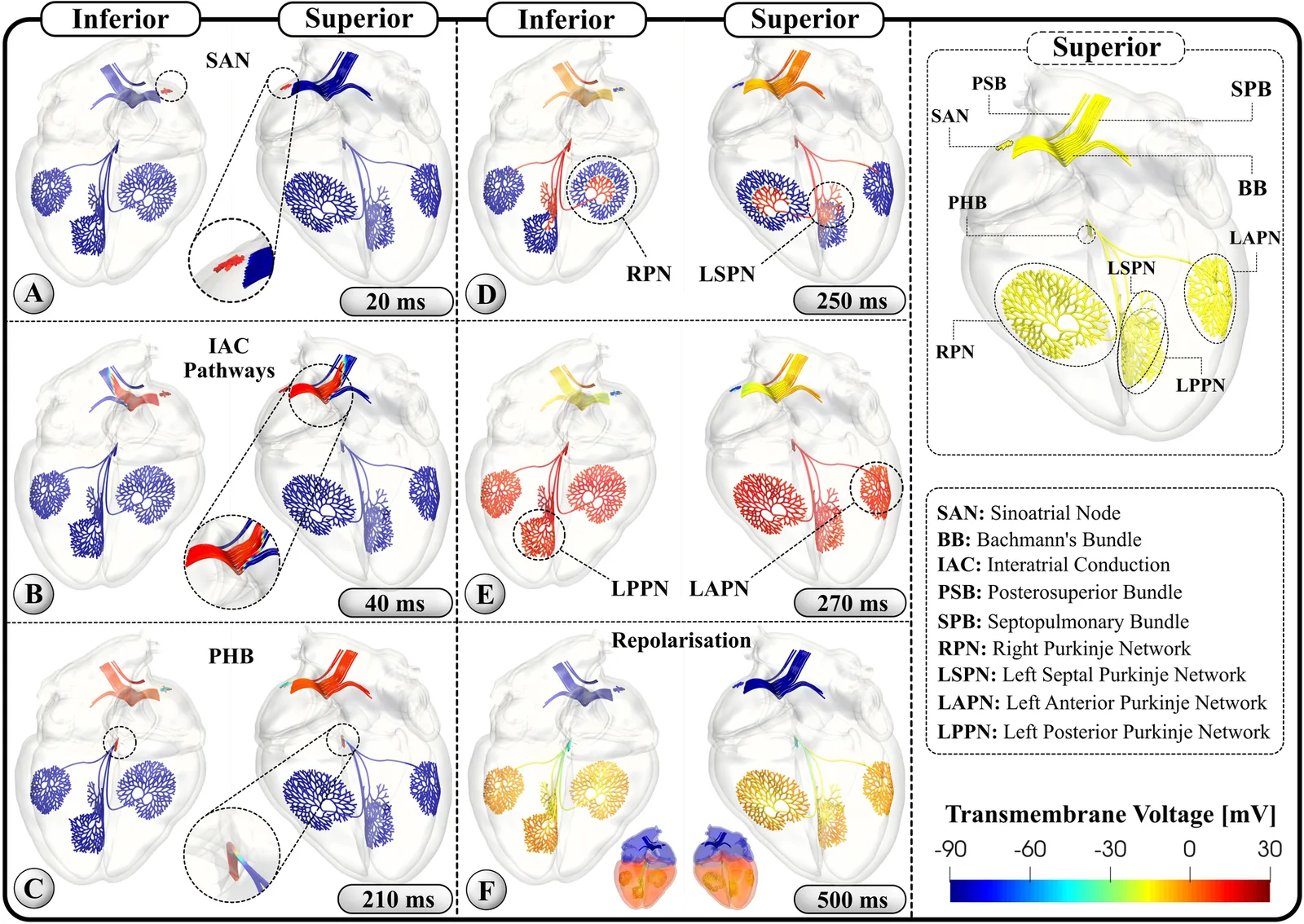
Simulated electrical activation sequence of the CCS and IAC in the human heart model. **(A)** Initiation of electrical activity within the SAN and propagation toward the atrial myocardium via the SAN exit pathways. **(B)** IAC mediated predominantly by the BB, with contribution from the SPB. **(C)** Activation of the PHB. **(D)** Activation of the PN in the RV and the septal PN in the LV. **(E)** Subsequent activation of the anterior and posterior PN in the LV. **(F)** Ventricular repolarisation.

Although electrical excitation reached the proximal portions of all bundle branches concurrently, differences in fascicular length and ventricular course resulted in staggered activation times at the respective Purkinje–myocardial junctions. Ventricular activation emerged first from the left septal fascicle (Figure 10D), followed sequentially by the anterior, posterior and RBB fascicles. Myocardial breakthrough occurred at approximately 30, 33, 35, and 40 ms after PHB activation, respectively (Figures 10D–F).

From these junctions, the PN rapidly distributed excitation across the ventricular endocardium, leading to near-synchronous activation of both ventricles.

Although RBB-mediated endocardial breakthrough occurred later than the LV fascicular breakthroughs, the earliest epicardial breakthroughs were observed nearly simultaneously on the RV free wall and at the anterior interventricular junction (Figure 11B), reflecting the shorter transmural distance across the thinner RV wall.

**Figure 11:**
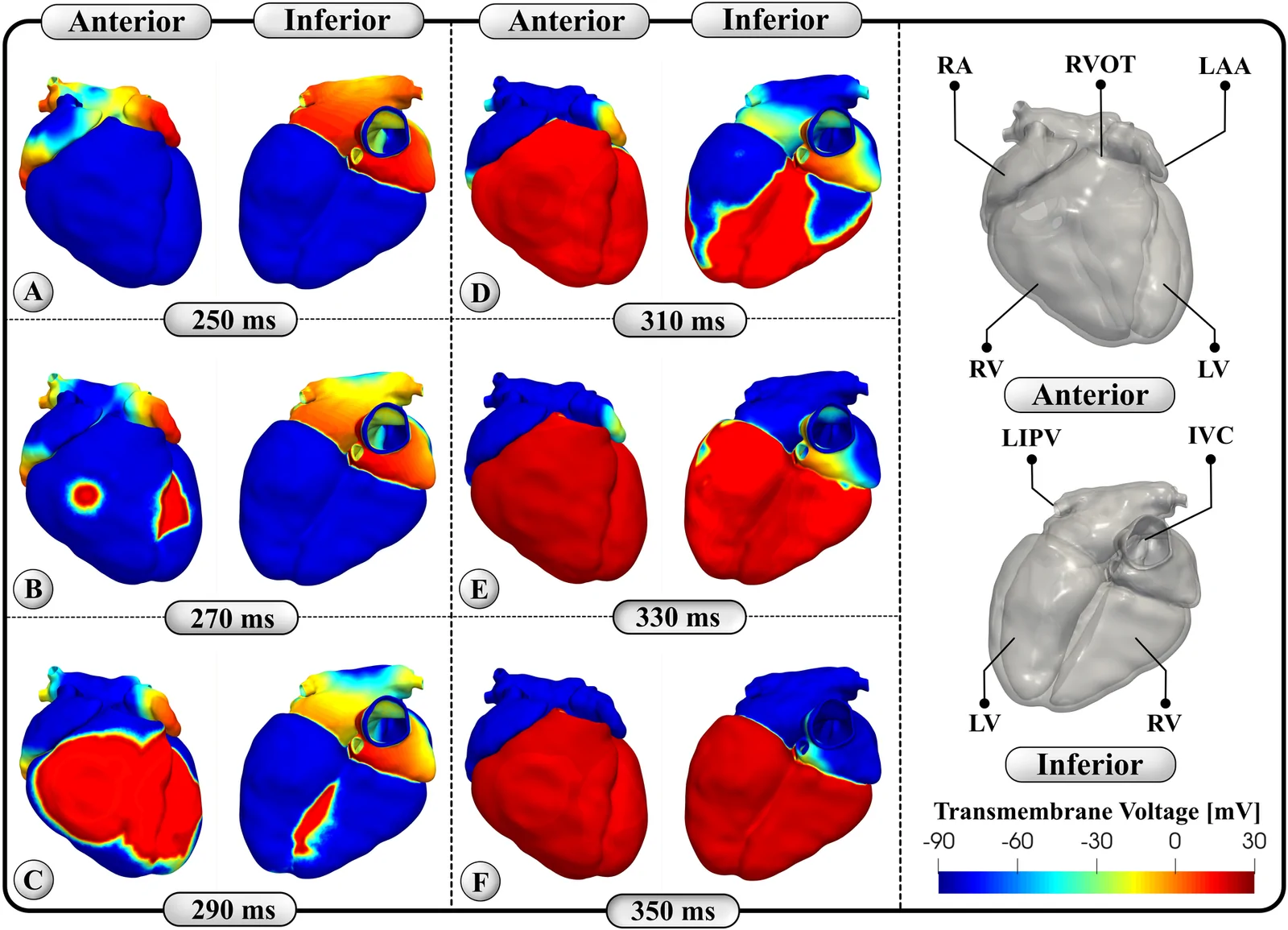
Simulated ventricular activation in the human heart model. **(A)** The earliest epicardial breakthrough was observed on the right ventricular free wall, mediated via the RBB–Purkinje system. **(B)** A subsequent breakthrough emerged along the anterior interventricular groove, reflecting early LV endocardial-to-epicardial activation mediated by the anterior and septal PN. **(C)** Additional posterior breakthroughs became evident as activation progressed through the posterior Purkinje branches. **(D–F)** Ventricular depolarisation then spread epicardially to complete activation, with the final regions activated located at the basal posterolateral wall of the LV and the posterior basal region of the RV.

Subsequently, activation propagated from the endocardium toward the epicardium. In the LV, two dominant epicardial wavefronts emerged—one originating from the anterior wall and the other from the posterior region (Figure 11C). These wavefronts progressed laterally and collided at the LV lateral wall, completing LV activation at the basal lateral wall (Figure 11D).

In the RV, activation originating from the free wall expanded posteriorly, while a second wavefront advanced from the posterior interventricular groove (Figure 11C). The collision of these wavefronts at the posterior right ventricular wall marked the completion of global ventricular activation (Figures 11E,F).

### 3.3 Electrocardiogram

Simulated 12-lead ECG demonstrated close agreement with the measured recordings, with stronger correspondence during ventricular depolarisation and overall agreement during atrial depolarisation. Quantitative comparison was performed using global P-wave and QRS windows together with lead-specific Pearson correlation analysis (Figure 12, Table 3). The measured and simulated P-wave durations were 71 and 98 ms, respectively. The corresponding isoelectric intervals were 138 and 112 ms, and the QRS durations were 69 and 79 ms. Across the 12 leads, the mean Pearson correlation was 0.73 for the P-wave and 0.91 for the QRS complex, indicating acceptable morphology agreement during atrial and ventricular depolarisation.

**Figure 12:**
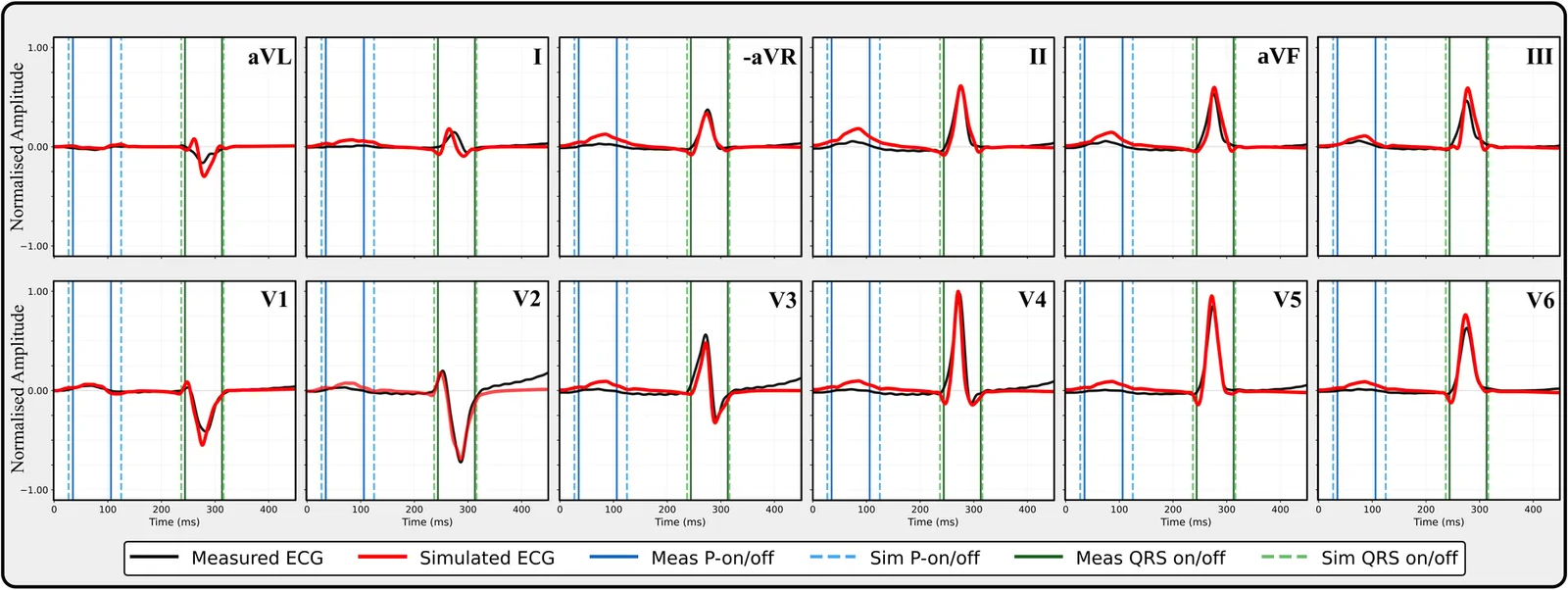
Comparison of normalised measured (black) and simulated (red) 12-lead ECG. Solid and dashed blue vertical lines indicate measured and simulated P-wave onset and offset, respectively. Solid and dashed green vertical lines indicate measured and simulated QRS onset and offset, respectively. The simulated ECG reproduced the main P-wave and QRS morphologies across the 12 leads, with lead-specific correlations reported in Table 3.

**Table 3:** Quantitative comparison between measured and simulated ECG features.

Metric	Measured	Simulated
P-wave duration	71 ms	98 ms
Isoelectric interval	138 ms	112 ms
QRS duration	69 ms	79 ms

Lead	P-wave r	QRS r
aVL	0.832	0.712
I	0.480	0.658
−aVR	0.851	0.974
II	0.822	0.960
aVF	0.808	0.919
III	0.804	0.855
V1	0.968	0.954
V2	0.541	0.988
V3	0.493	0.981
V4	0.587	0.961
V5	0.756	0.986
V6	0.779	0.991
Mean	0.727	0.912

Pearson correlation coefficients (r) were computed separately for the P-wave and QRS complex in each lead.

## 4 Discussion

This study presents an anatomy-guided, computational model of the human CCS embedded within a subject-specific whole-heart electrophysiological framework.

By integrating the SAN, major IAC pathways, the AVN, and the His–Purkinje system into a unified myocardial geometry, the model enabled whole-heart simulation from sinus initiation to ventricular depolarisation. In contrast to previous computational representations that focused on isolated components or partial subsystems of the CCS (108–110), the present framework incorporates all principal conduction structures within a single anatomically consistent model, thereby facilitating organ-scale analysis of activation sequences and their manifestation on the surface ECG.

### 4.1 Atrial activation and interatrial conduction

Physiological atrial activation originates from the SAN, a structurally heterogeneous three-dimensional nodal complex coupled to the surrounding atrial myocardium through discrete SAN exit pathways and embedded within preferential conduction substrates such as the CT and BB (30, 36, 43). Although these mechanisms have been extensively characterised experimentally, their integration within subject-specific computational models remains limited. Most existing frameworks simplify nodal structure or treat atrial pathways in isolation, whereas integrated reconstruction of the SAN and IAC within a unified organ-scale model is rarely achieved (13, 39, 111).

Atrial activation was initiated within a centrally located leading pacemaker region of the SAN and transmitted to the RA via anatomically defined SAN exit pathways. This configuration enabled a right atrial breakthrough pattern characterised by early activation along the CT, followed by preferential longitudinal propagation toward the superior and inferior vena cava and transverse spread toward the IAS, consistent with clinically observed activation sequences (35, 43, 112). Rather than modelling emergent pacemaker automaticity, the three-dimensional SAN provides an anatomically grounded substrate for nodal geometry, insulation, and exit-pathway coupling, serving as a blueprint for future studies requiring more detailed investigation of SAN function. IAC in the model was mediated predominantly by the BB, in line with anatomical and functional evidence identifying it as the principal pathway for left atrial activation during sinus rhythm (51, 52). Breakthrough at the superior left atrial dome via the SPB, followed by a secondary activation site near the LAA, reflects the branching and anterior course of the BB. The PSB contributed only minimally to IAC, which was attributed to the early recruitment of lateral SAN exit pathway located in close proximity to the BB. Alternative posterior routes of IAC may assume greater functional significance when anterior pathways are less dominant or functionally impaired (53). Completion of atrial depolarisation through wavefront collision at the left atrial isthmus further supports the physiological coherence of the simulated activation sequence and mirrors observations from high-resolution clinical mapping studies (43).

The electrophysiological properties assigned to the atrial components, particularly regional conduction velocities and anisotropy, were tuned to ensure synchronous atrial depolarisation and to reproduce a P-wave morphology and duration consistent with the measured recording. However, with the semi-auto calibration, lower correlations remained in selected leads, particularly the anterior precordial leads. A more comprehensive parameter-calibration strategy may further improve lead-specific agreement.

For instance, a conduction velocity of 2.0 m/s was assigned to the cables representing the BB. Although this value is slightly higher than reported physiological measurements (113), it was selected to compensate for the simplified representation of the BB, in which intramural branches were omitted, and to improve the overall P-wave morphology. This global improvement may nevertheless come at the expense of morphology agreement in individual leads, potentially contributing to the lower correlations observed in some precordial leads.

In addition to the major pathways described above, anatomical structures such as the CT and the rim of the FO contribute to IAC and modulate atrial activation patterns (114). Incorporating these substrates within a coherent structural and electrophysiological framework is therefore essential for reproducing realistic atrial activation sequences in whole-heart computational models.

### 4.2 Atrioventricular nodal activation

The AVN represents one of the most anatomically and functionally intricate components of the CCS. While its gross anatomy is well characterised (115), the mechanisms governing atrioventricular conduction under varying conditions – specifically under pathological conditions – remain complex (116). Experimental and mapping studies indicate that, under normal sinus rhythm, atrioventricular conduction arises from the convergence of two distinct atrial wavefronts at the atrioventricular junction (117). One wavefront propagates medially from the IAS toward the ToK via the FP. A second wavefront travels through the inferior RA and tricuspid isthmus via the SP. These wavefronts converge within the ToK, with the earliest arrival engaging the CN. Subsequent activation within the AVN is slow and spatially confined, ensuring an atrioventricular delay within the physiological range of approximately 60–80 ms before conduction to the HB (57, 68, 117–119). Importantly, the earlier arrival through the FP reflects its shorter overall anatomical route and does not necessarily imply a higher local conduction velocity than in the SP (57).

The human CN can give rise to two extensions: right inferior nodal extension (RINE), which courses along the septal tricuspid leaflet and can reach the coronary sinus ostium, and the shorter left inferior nodal extension (LINE) originating near the IAS (60, 120–122), both converging proximally to form the CN base (121, 122). Nodal extensions were not explicitly implemented. Instead, the dual atrionodal inputs were represented by an anterior FP associated with transitional tissue approaching the CN, and an inferior–posterior SP entering the nodal complex via the tricuspid isthmus, consistent with the anatomical construction described in Section 2.1.3. Furthermore, the functional behaviour of the pathways depends on the interplay between pathway geometry, conduction time, and refractoriness. A thorough demonstration of this behaviour would require dedicated premature-stimulation protocols and functional characterisation, which were beyond the scope of the present study.

Conduction slowing within the AVN arises from multiple factors—primarily low expression of connexins, particularly Cx43, which results in sparse gap-junctional coupling, and reduced sodium channel density, which further limits impulse propagation (121–123). These factors, among others, underlie extremely slow, yet reliable, conduction, with velocities as low as 2 cm/s (118). AVN anatomy and conduction velocity underpin its electrophysiological role in gating and maintaining the atrioventricular delay. In computational models, physiologically consistent atrioventricular conduction depends on appropriate parameterisation of AVN geometry and electrophysiological properties, as variations in these parameters can influence atrioventricular delay and the timing of His-bundle activation.

### 4.3 Ventricular activation

A trifascicular configuration of the LBB was implemented within the LV, together with a single RBB trunk supplying the RV, in accordance with commonly described anatomical patterns (83, 84, 124). Although bundle branches exhibit substantial interindividual variability in their branching architecture and spatial course, a trifascicular configuration of the LBB is widely reported in anatomical and clinical studies and is frequently regarded as the prevalent configuration (83, 125, 126). In contrast, the organisation of the RBB remains less clearly defined. While classical descriptions have suggested the presence of septal (127) and posterior subdivision (128), recent anatomical investigations emphasise that the RBB may remain electrically insulated along much of its intraseptal course, emerging predominantly near the base of the anterior papillary muscle in the RV (81, 124). This remains debated, particularly regarding whether the RBB provides direct septal or medial papillary inputs. Accordingly, the present representation should be interpreted as one anatomically plausible configuration rather than a definitive anatomical template.

Beyond reproducing ventricular activation sequences consistent with classical human mapping studies (127, 129, 130), this framework underscores the sensitivity of ventricular depolarisation to both the geometric configuration and electrophysiological properties of the His–Purkinje system. Variations in the length, spatial trajectory, and branching pattern of the main fascicular trunks, together with regional differences in conduction velocity, directly modulate the timing of endocardial breakthrough and the subsequent epicardial activation sequence. Such dependencies are well documented in anatomical and clinical investigations, where even subtle differences in bundle branch or fascicular architecture have been associated with measurable alterations in activation patterns and QRS morphology (80, 131, 132).

In addition to the proximal fascicular trunks, the distal PN substantially shapes ventricular activation by governing the spread of excitation following endocardial breakthrough. The regional ramification topology of the distal PN exhibits pronounced inter-individual variability in both density and spatial organisation (133), and cannot be resolved from available imaging data. A surrogate representation was therefore adopted: rather than imposing a single homogeneous fast subendocardial layer (17, 134), the ventricular subendocardium was partitioned into anterior, septal, and posterior regions assigned regionally differentiated isotropic conduction velocities, calibrated jointly to reproduce the measured ECG. This regionalised surrogate acknowledges the reported regional heterogeneity of distal PN density (110, 133) while avoiding speculative topology reconstruction.

### 4.4 ECG agreement and consistency assessment

Forward ECG modelling now routinely reproduces body-surface potentials from simulated cardiac electrical activity (107, 135). However, calibrating model parameters to measured ECG data remains inherently ill-posed (136), as the high dimensionality of the parameter space allows multiple physiologically distinct configurations to generate similar ECG morphologies (137, 138). This non-uniqueness was further demonstrated by Cicci et al. (139), who considered 104 anatomical and electrophysiological parameters and showed that QRS morphology was mainly controlled by myocardial conduction velocity, fast subendocardial conduction velocity, conduction-velocity gradients, fascicle/root locations, the right-ventricular moderator-band root, and blood conductivity. Consequently, agreement between simulated and measured ECGs, while necessary, does not uniquely determine the underlying activation sequence. The anatomical and electrophysiological constraints therefore support physiological consistency across the complete conduction axis, rather than demonstrating reduced calibration ambiguity.

The close correspondence between simulated and measured 12-lead ECG demonstrates that the anatomy-guided CCS framework is consistent with the measured ECG phenotype. Therefore, the calibrated conduction velocities should not be interpreted as a unique parameter set, but as one physiologically plausible solution constrained by anatomy, literature-based parameter ranges, and the measured ECG. In this context, ECG agreement serves not merely as a fitting target but as a functional consistency check of the integrated anatomical–biophysical representation.

### 4.5 Hybrid 1D-3D modelling strategy

In this study, one-dimensional cable representations were employed to provide an anatomical substrate for rapid conduction in the IAC and His-Purkinje network, without introducing discrete tracts into the mesh. This approach preserves conduction continuity consistent with experimental observations (48, 49) while reducing computational cost. In contrast, regions in which one-dimensional cable representations were insufficient to capture the underlying structural and electrophysiological complexity—particularly nodal components (SAN and AVN)—were reconstructed as volumetric structures and fully integrated into the myocardial mesh. This strategy enabled representation of their three-dimensional architecture, slow conduction properties, and complex coupling with surrounding tissue, thereby preserving physiological fidelity in areas where simplified cable abstractions would not adequately reproduce their behaviour.

### 4.6 Limitations and future direction

The present study has several limitations. First, the geometry of the CCS was reconstructed from contemporary anatomical, histological, and functional descriptions, but it cannot capture the full spectrum of inter-individual variability in human conduction system anatomy. Variations in the size, branching pattern, spatial orientation, and myocardial coupling of the SAN, IAC pathways, AVN, and His–Purkinje system are well documented, whereas subject-specific reconstruction of these structures remains infeasible with current in vivo imaging. The model should therefore be interpreted as an anatomy-guided and physiologically plausible representation, rather than a definitive subject-specific reconstruction of the CCS. For the same reason, transfer of the framework to another patient mesh is feasible, but currently requires manual or semi-manual landmark annotation and expert quality control. Beyond this, automated transfer between different anatomical meshes would be feasible using anatomical reference frames, such as universal ventricular and atrial coordinates (UVC and UAC), although this has not been tested here.

Second, electrophysiological parameters were assigned regionally from literature values and subsequently adjusted to improve agreement between simulated and measured ECGs. Although this approach is common in whole-heart modelling and produced activation timings within reported physiological ranges (57, 127, 140, 141), it does not replace subject-specific electrophysiological measurements. Parameter tuning therefore introduces non-uniqueness, whereby multiple parameter combinations may reproduce similar ECG features. More, the ECG-based calibration framework used in this study constitutes a separate methodological development beyond the scope of the present manuscript. A detailed description and evaluation of the method will be provided in a dedicated modelling study currently in preparation.

Third, the AVN was represented using available atrial ionic formulations and region-specific conduction properties, as a fully validated human AVN-specific ionic model suitable for whole-heart simulations remains unavailable. Consequently, the model approximated the macroscopic delay function of the AVN, including delayed atrioventricular conduction, but did not resolve the full cellular heterogeneity of nodal, transitional, and nodal–Hisian tissue.

In the ventricles, the PN was represented by primary fascicular networks anchored at anatomically motivated terminal regions, serving as the initial endocardial breakthrough sites for each fascicle. The remaining ramifications, whose regional topology and density cannot be resolved from available imaging data, were replaced by a regionalised subendocardial surrogate with differentially assigned isotropic conduction velocities. Although this strategy reproduced ventricular activation patterns consistent with classical mapping studies (127), it does not capture the full anatomical complexity of the PN or its potential role in fine-scale activation heterogeneity. QRS morphology is expected to be sensitive to the location of fascicular roots, the extent of fast subendocardial activation, and the assigned fast endocardial conduction velocities. This sensitivity has been demonstrated in related ECG-calibration studies, including Cicci et al. (139), but a systematic sensitivity analysis of these parameters was outside the scope of the present anatomy-guided conduction-system reconstruction.

Finally, the present framework was designed for efficient activation modelling using a reaction–eikonal formulation. This choice is supported by recent work showing that the same physics-informed eikonal approach can reproduce whole-heart electrophysiology with near real-time performance while maintaining close agreement with a gold-standard electrophysiology model (142). The fine geometric resolution required for volumetric nodal regions may, however, limit direct transfer of the full model to conventional reaction–diffusion simulations without additional mesh adaptation or numerical considerations. Moreover, repolarisation dynamics were not systematically investigated; future work should assess how CCS architecture, PN topology, and subendocardial conduction surrogates influence repolarisation patterns and ECG repolarisation indices.

### 4.7 Implications and future applications

This work provides a unified whole-heart framework in which all major CCS components are explicitly represented. By integrating the SAN, interatrial pathways, AVN, His–Purkinje system, and a regionally differentiated ventricular subendocardial conduction substrate within a unified geometry, the model enables systematic investigation of how conduction system structure and regional electrophysiological properties govern global activation patterns and surface ECG morphology.

The framework is particularly suited for mechanistic studies of conduction system pacing, atrioventricular conduction abnormalities, and bundle branch block phenotypes, where small variations in conduction pathway engagement can markedly alter ventricular activation. From a research perspective, the model offers a controlled environment for testing hypotheses on conduction system anatomy–function relationships that are difficult to isolate experimentally. Clinically, it enables in silico evaluation of pacing strategies and lead placement, relevant for therapy planning and interpretation of complex activation patterns.

## Data Availability

The raw data supporting the conclusions of this article will be made available by the authors, without undue reservation.
